# Myocardial Viability and Ischaemia in Chronic Total Occlusion

**DOI:** 10.3390/medicina62030540

**Published:** 2026-03-13

**Authors:** Zia Mehmood, Preethi Suresh, Rui Li, Hosamadin Assadi, Bahman Kasmai, Kurian Thampi, Clint Maart, Timothy Gilbert, Simon Eccleshall, Chris Sawh, Sunil Nair, Rob J. van der Geest, Vassilios S. Vassiliou, Alisdair Ryding, Gareth Matthews, Pankaj Garg

**Affiliations:** 1 Department of Cardiology, Norfolk and Norwich University Hospitals NHS Foundation Trust, Norwich NR4 7UY, UK; preethi.suresh3@nhs.net (P.S.); r.li2@uea.ac.uk (R.L.); simon.eccleshall@nnuh.nhs.uk (S.E.); chris.sawh@nnuh.nhs.uk (C.S.); sunil.nair@nnuh.nhs.uk (S.N.);; 2 Department of Cardiovascular and Metabolic Health, Norwich Medical School, University of East Anglia, Norwich NR4 7UQ, UK; 3 Department of Radiology, Division of Image Processing, Leiden University Medical Center, 2333 ZG Leiden, The Netherlands

**Keywords:** chronic total occlusion, cardiac magnetic resonance imaging, myocardial viability, myocardial ischaemia, late gadolinium enhancement

## Abstract

*Background and Objectives:* Chronic total occlusion (CTO) affects 30% of patients undergoing coronary angiography rendering poorer outcomes. While percutaneous coronary intervention (PCI) can be technically successful, RCTs show no survival benefit. Cardiovascular Magnetic Resonance (CMR) provides comprehensive myocardial phenotyping, offering prognostic insights in this high-risk cohort. *Materials and Methods:* Fifty-six patients with angiographically confirmed CTO underwent stress perfusion CMR with late gadolinium enhancement. Myocardial function, ischaemia and scar burden were quantified and compared across CTO territory and viability subgroups. *Results*: In patients with CTO, 27% of patients (15/56) had no viability. In patients with viable myocardium, 66% (27/41) demonstrated reversible ischaemia. Viable myocardium was associated with significantly higher LV stroke volumes (93.6 ± 20.1 mL vs. 80.9 ± 18.4 mL, *p* = 0.039), along with lower LV scar mass (18.7 ± 13.5g vs. 32.3 ± 12.8g; *p* = 0.002) and scar percentage (14.9 ± 8.3% vs. 25.9 ± 7.5%; *p* = 0.001). Viable myocardium showed more ischaemia both globally (11.6 ± 14.3g vs. 0.2 ± 9.3g; *p* = 0.005) and within the CTO territory (10.3 ± 10.3% vs. 2.3 ± 2.7%; *p* = 0.01). Non-viable myocardium was associated with significantly higher CTO-territory scar mass (9.4 ± 6.5 g vs. 5.1 ± 6.9 g; *p* = 0.046) and scar percentage (21.8 ± 13.3% vs. 11.7 ± 12.8%; *p* = 0.01), indicating extensive fibrosis. A scar burden threshold of 11.18% in CTO territory predicted non-viability with 80% sensitivity and 65.85% specificity (AUC = 0.701 [95% CI 0.54–0.87], *p* = 0.019). *Conclusions***:** Among CTO patients, 27% harbour no viability, while patients with viable myocardium typically exhibit reversible ischaemia—representing a phenotype with preserved viability and inducible ischaemia. These findings support the use of multiparametric CMR to phenotype CTO territories prior to considering CTO-PCI.

## 1. Introduction

Chronic total occlusion (CTO) is defined as a complete coronary artery blockage persisting for over two to three months, affecting 20–30% of patients undergoing coronary angiography [1,2,3,4]. Compared to non-obstructive coronary artery disease (CAD), CTO is associated with adverse clinical outcomes and increased mortality, particularly in patients with left ventricular (LV) dysfunction [1,5]. While technical success rates for PCI have improved significantly, the prognostic benefit of CTO-PCI remains uncertain due to conflicting results across randomised controlled trials (RCTs) and observational studies [6,7,8,9,10,11].

Current clinical guidelines advocate for CTO-PCI primarily to alleviate symptoms and reduce myocardial ischaemia, yet patient selection strategies remain imprecise [5]. Traditional decision-making has relied heavily on anatomical data—such as occlusion length, collateral grade, or target vessel territory—without consistent incorporation of myocardial functional data [3,4,5,7,12]. This approach may overlook the substantial heterogeneity in myocardial response to chronic occlusion, including variations in scar burden, ischaemic burden, and viability, which have critical implications for recovery potential and long-term benefit following PCI [13,14,15]. Contemporary myocardial revascularisation guidance emphasises integrating objective evidence of ischaemia and/or viability when considering CTO revascularisation [5].

However, CTO-PCI trials have enrolled heterogeneous populations without systematic multiparametric phenotyping of the scar and inducible ischaemia, which may contribute to neutral outcomes and ongoing uncertainty in patient selection. Accordingly, an imaging strategy that integrates ventricular function, scar burden, and inducible ischaemia may help identify potentially recoverable myocardium and avoid revascularisation of extensively scarred territories.

Stress perfusion CMR imaging has emerged as a key modality for advanced myocardial phenotyping, offering prognostic insights extending beyond conventional clinical risk factors in patients with suspected or established CAD [16,17,18]. Importantly, the detection of ischaemia on CMR stress perfusion is independently associated with an increased risk of adverse cardiovascular outcomes [16,17,19]. Although invasive coronary angiography remains the gold standard for identifying CTO, it provides no information about myocardial ischaemia, infarction, and LV functional impairment [14,15]. In contrast, CMR offers a comprehensive non-invasive evaluation of myocardial perfusion, contractile function, and viability in a single scan. Stress CMR evaluates ischaemia during pharmacologic hyperaemia [20,21], and quantitative techniques enable precise measurement of myocardial perfusion reserve [22,23]. Moreover, CMR remains the reference standard for characterising myocardial scar via late gadolinium enhancement (LGE) and regional LV function [24,25].

Despite these advantages, the application of quantitative stress perfusion and LGE imaging for phenotyping CTO patients remains underutilised. Angiographic measures, such as collateral flow, have traditionally been used to estimate myocardial viability, but emerging data suggest CMR provides superior risk stratification in predicting viability [26]. Furthermore, the discrepancies between RCTs and observational studies regarding the prognostic impact of CTO-PCI may stem from challenges in randomising this patient population, largely due to the absence of consistent functional imaging-based phenotyping [12,24,27,28]. This underscores the need for more personalised, mechanistic approaches to patient selection based on detailed myocardial assessment. This study aims to characterise and advance the understanding of myocardial ischaemia and myocardial scar burden in patients with CTO through comprehensive CMR-based evaluation.

## 2. Methods

### 2.1. Study Cohort

We conducted a single-centre observational cohort study at Norfolk and Norwich University Hospitals NHS Foundation Trust, identifying patients with documented CTO who underwent CMR for myocardial viability assessment between 2016 and 2021; 56 patients met the inclusion criteria. Eligibility criteria included: (1) age between 18 and 90 years; (2) diagnosis of stable or unstable angina; (3) invasive coronary angiography confirming chronic total occlusion in at least one major coronary artery (left anterior descending, left circumflex, or right coronary artery); and (4) completion of a stress perfusion CMR scan. Exclusion criteria were: (1) contraindications to CMR or known gadolinium allergy; (2) hospitalisation for acute myocardial infarction within 90 days before the CMR; (3) presence of non-ischaemic heart disease (including congenital heart disease or severe valvular disease); and (4) advanced chronic renal failure.

CTO was defined as a complete blockage of the coronary artery on invasive coronary angiogram with no blood flow beyond the occlusion (TIMI grade 0) for a duration exceeding three months. Multi-vessel disease was classified as the presence of severe stenosis (≥70%) in at least one additional artery apart from the CTO vessel. CTO duration was determined from prior angiography where available; otherwise, duration was inferred from clinical history (symptom onset or prior myocardial infarction) and angiographic features, consistent with standard CTO definitions.

### 2.2. Ethics Approval and Consent to Participate

The study adhered to the Declaration of Helsinki (2013) and was conducted under the favourable opinion of the Faculty of Medicine and Health Sciences Research Ethics Committee, University of East Anglia (Cardiovascular Imaging Registry for Clinical Improvement [CIRI]; Ref. 2020/21-075), within the UEA-approved CIRI database. In line with REC guidance, individual informed consent was waived for secondary (retrospective) analysis of routinely collected clinical data, on the basis that the research introduced no intervention or alteration to clinical care, and all processing occurred within the host NHS organisation; identifiable patient-level data did not leave the organisation, and analyses were performed on de-identified datasets. The study is registered on ClinicalTrials.gov under registration number NCT05114785.

### 2.3. Coronary Angiogram Analysis

Invasive coronary angiographic data was independently reviewed in a blinded fashion by experienced cardiology fellow P.S. The analysis focused on confirming the presence of CTO, including lesion location in left anterior descending (LAD), left circumflex artery (LCx), and right coronary artery (RCA) as well as bystander coronary disease. Blinding of the reviewer to clinical and imaging data minimised potential bias and enhanced the reliability of lesion assessment and interpretation of angiographic findings.

### 2.4. CMR Protocol

All examinations were performed using a 1.5 T scanner (Siemens Healthineers, Erlangen, Germany). Prospective ECG gating was applied using the vendor’s standard vector-ECG algorithm. All perfusion data were acquired during free breathing. All patients had intravenous access for both adenosine infusion and contrast administration, in line with standard practice at the time.

The CMR protocol included balanced steady-state free precession (bSSFP) baseline cines (4-chamber, 2-chamber, short-axis left ventricular stack) with 30 phases throughout the cardiac cycle, first-past perfusion post adenosine intravenous infusion and LGE imaging for scar assessment. 

Stress perfusion was performed with a single-bolus, single-sequence first-pass protocol. Hyperaemia was induced with an intravenous infusion of adenosine (AAH Pharmaceuticals Ltd., Warwick, UK) at 140 µg kg^−1^ min^−1^ for four minutes. A single undiluted bolus of gadobutrol 1.0 mmol mL^−1^ (Gadovist^®^, Bayer AG, Leverkusen, Germany) was injected at 0.05 mmol kg^−1^ through the antecubital vein at 3 mL s^−1^ immediately after 150 s of adenosine infusion, followed by a 20 mL saline flush (BD^®^ PosiFlush™, Berkshire, UK). No preparatory dilute pre-bolus or dual-sequence acquisition was used; hence the arterial input function and myocardial first-pass were captured within the same saturation-recovery gradient-echo read-out. Image acquisition commenced after 150 s of infusion once haemodynamic steady state was confirmed. Stress adequacy was defined by an increase in heart rate of at least ten beats per minute and/or a fall in systolic blood pressure of ten millimetres of mercury, or by the appearance of typical vasodilator symptoms. All scans included in the analysis met these stress adequacy criteria.

Myocardial perfusion was imaged with a single-shot saturation-recovery gradient-echo sequence (TurboFLASH) accelerated with GRAPPA (R = 2). Three short-axis slices at basal, mid-ventricular and apical levels were collected every cardiac cycle, giving an in-plane spatial resolution of 2.7 × 2.2 mm^2^, slice thickness 8 mm, repetition/echo times of 2.6/1.1 ms, flip angle 12°, and an effective saturation-recovery time of 105 ms. The acquisition bandwidth was 651 Hz px^−1^ and the temporal footprint per slice was 188 ms. In total 60 dynamics were acquired during the first-pass acquisition.

Rest perfusion imaging was acquired after cessation of adenosine and an appropriate washout period (minimum 10 min), using the same sequence parameters and a second bolus of gadobutrol 0.05 mmol kg^−1^ (Gadovist^®^, Bayer AG, Leverkusen, Germany) followed by a 20 mL saline flush (BD^®^ PosiFlush™, Berkshire, UK).

LGE was acquired six to ten minutes after completion of rest perfusion with a two-dimensional phase-sensitive inversion-recovery segmented gradient-echo sequence. Contiguous short-axis slices covering the entire left ventricle together with standard two-, three- and four-chamber long-axis views were obtained during end-expiratory breath-holds under prospective ECG gating. Typical acquisition settings were a repetition time of 2.93 ms, an echo time of 1.12 ms, a flip angle of 45°, and an in-plane spatial resolution of 1.9 × 1.4 mm^2^ reconstructed to 1.4 × 1.4 mm^2^. Slice thickness was fixed at 8 mm with no inter-slice gap, the field of view was 300–360 mm, and the receiver bandwidth was 781 Hz px^−1^. Immediately before each acquisition, a Look-Locker inversion-time scout was performed and the inversion time was adjusted to null normal myocardium—typically between 260 ms and 320 ms at 1.5 T. Phase-sensitive reconstruction was used to preserve myocardial–blood contrast in the presence of heart rate variability, and magnitude and phase images were reconstructed inline and exported in DICOM format for off-line quantitative scar analysis.

### 2.5. CMR Image Analysis and Quantitative Post-Processing

Comprehensive post-processing of cine, LGE and first-pass perfusion datasets was conducted using the semi-automated in-house developed MASS research platform (Version 2022-EXP; Leiden University Medical Center, Leiden, The Netherlands) [29]. All analyses were performed on short-axis image stacks encompassing the entire LV, from base to apex, across the full cardiac cycle. Papillary muscles were excluded from the myocardial mask to avoid overestimation of myocardial mass and underestimation of scar burden. For anatomical consistency across all imaging sequences, the insertion points of the right ventricular (RV) free wall into the interventricular septum were identified and aligned on basal short-axis slices. The semi-automated contouring and image analysis were performed by senior cardiology fellow Z.M. under direct supervision of P.G. (Level III-certified CMR expert—EACVI/SCMR). All segmentations and parametric data extractions were independently reviewed and validated by P.G. to ensure methodological rigor and consistency.

### 2.6. Myocardial Segmentation and Regional Framework

For regional myocardial assessment, the LV myocardium was segmented according to the standardised 16-segment model proposed by the American Heart Association (AHA) [30]. This segmentation framework was uniformly applied across LGE, and perfusion sequences, enabling integration of the tissue characterisation and perfusion data. Bull’s-eye plots were generated to facilitate intuitive visualisation and segment-level comparison. CTO territory segments were assigned to the occluded vessel using standard coronary perfusion territories, with adjustment for angiographic dominance.

### 2.7. Quantification of Myocardial Scar (LGE)

LGE was analysed on phase-sensitive inversion recovery (PSIR) images. Areas of fibrosis were identified as regions with signal intensity exceeding five standard deviations above the mean of a remote, unaffected myocardial reference region. The region-of-interest (ROI) was placed meticulously in remote myocardium, avoiding areas of partial volume effects or suspected LGE. Segmental quantification of LGE burden was performed semi-automatically on all short-axis slices, spanning from the mitral valve annulus to the apex. Scar burden was quantified in grams as well as percentage and mapped to the AHA 16-segment model, facilitating regional stratification of fibrotic involvement.

### 2.8. Myocardial Perfusion Analysis

First-pass perfusion CMR was analysed qualitatively during adenosine-induced pharmacologic stress. Stress perfusion defects were assessed on a segmental basis, co-registered to the 16-segment AHA framework. Perfusion analysis was performed collaboratively by P.G. and Z.M. to ensure interpretative consistency. Perfusion defects were identified as regions demonstrating a reduction in myocardial blood flow during stress relative to rest. Reversible perfusion (ischaemic) defects were defined by the presence of stress-induced hypoperfusion in segments without corresponding LGE or with ≤50% LGE transmurality. Total and regional myocardial perfusion defect mass were quantified (in grams) as previously described [31]. The reversible perfusion defect was quantified by subtracting the LGE-defined scar mass from the total perfusion defect mass. Perfusion-defect mass (g) was derived by summing the myocardial mass of AHA segments demonstrating visually apparent stress hypoperfusion, as implemented in the MASS platform. Disagreements were resolved by consensus; formal interobserver reproducibility testing was not performed.

### 2.9. Myocardial Viability Classification

Myocardial viability within the CTO territory was determined through the integrated assessment of LGE extent and perfusion in accordance to current practice [32]. Segments with LGE involving ≤50% of the myocardial wall thickness were considered viable. In contrast, segments demonstrating LGE exceeding 50% of the wall thickness were classified as non-viable [32]. At the patient level, CTO territory viability was defined by the presence of at least one CTO-territory segment with ≤50% LGE transmurality; territories with all segments demonstrating >50% transmural LGE were classified as non-viable.

### 2.10. Extracted Quantitative Parameters

These quantitative measures provided a comprehensive evaluation of myocardial structure, perfusion, and viability, allowing for detailed characterisation of ischaemic and fibrotic burden within the CTO-perfused myocardium.

1. Ventricular Volumes, Function, and Mass (Cine images)
  LV and RV end-diastolic volume (EDV) and end-systolic volume (ESV)
  LV and RV ejection fraction, calculated as (EDV−ESV)/EDV × 100
  Stroke volume (SV), defined as (EDV−ESV)
  LV myocardial mass, excluding papillary muscles
  These indices offer a global assessment of ventricular remodelling and systolic function.
2. Myocardial Scar Burden (LGE)
  Total LV scar burden: Total LGE-derived fibrotic tissue mass in the LV (in grams and percentage)
  Regional scar burden: Scar mass and percentage within the CTO territory
  These parameters reflect both global and regional fibrotic remodelling and inform revascularisation decision-making.
3. Perfusion Defect Extent and Ischaemic Burden
  Reversible perfusion defect (ischaemia): Total perfusion defect mass minus scar mass (gram)
  Percentage of ischemic myocardium: Perfusion defect percentage minus scar percentage
  These provide an index of hypo-perfused yet viable tissue in the overall LV as well as the CTO territory, crucial for evaluating revascularisation benefit.
4. Viability Classification

Based on integration of LGE transmurality and perfusion defect analysis, enabling segment-level viability mapping.

### 2.11. Statistical Analysis

Statistical analyses were carried out using MedCalc^®^ Statistical Software (version 20.215; MedCalc Software Ltd., Ostend, Belgium) and OriginPro (version 2023; OriginLab Corporation, Northampton, MA, USA). The distribution of continuous variables was assessed for normality using the Shapiro–Wilk test. Results for continuous data are reported as mean  ±  standard deviation (SD), with comparisons between two independent groups made using the independent samples *t*-test. Categorical variables are summarised as frequencies and percentages. For comparisons involving more than two groups, one-way analysis of variance (ANOVA) was applied. The discriminative performance of variables was assessed by calculating the area under the receiver operating characteristic (ROC) area under curve (AUC). All statistical tests were two-tailed, with a *p*-value of less than 0.05 considered indicative of statistical significance. Additionally, 95% confidence intervals were reported for ROC analyses.

## 3. Results

### 3.1. Patient Characteristics

Baseline clinical and demographic characteristics of the study population are summarised in Table 1. A total of 56 patients with angiographically confirmed CTO of at least one coronary artery were included. The cohort was predominantly male (88%), with a mean age of 70.7 ± 10.2 years. The average height and weight were 171.8 ± 8.9 cm and 86.8 ± 15.7 kg, respectively. Prevalent cardiovascular risk factors in this population included chronic kidney disease (43%), dyslipidaemia (52%), diabetes mellitus (36%), hypertension (48%), and prior myocardial infarction (77%). Additional comorbidities included smoking history (66%), atrial fibrillation (14%), cerebrovascular accident (7%), and prior ventricular tachycardia (13%). Our patient cohort had a significant symptoms burden with a mean New York Heart Association (NYHA) functional class of 2.2 ± 1 and a mean Canadian Cardiovascular Society angina class of 3 ± 0.7. Comparative analysis between CTO territory sub-groups revealed no statistically significant differences in baseline characteristics.

### 3.2. Functional CMR Parameters and Myocardial Viability

**Figure 1:**
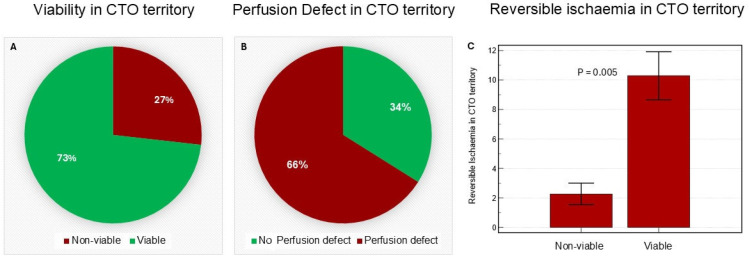
(**A**) Shows the percentage of patients with viable myocardium in the CTO territory. (**B**) Illustrates the proportion with reversible perfusion defects. (**C**) Displays the mean ± SD percentage of reversible ischaemia in the CTO territory among those with viable myocardium. Statistical differences were assessed using an independent *t*-test (*p*-values reported).

Of the total study cohort, 73% (*n* = 41) demonstrated viable myocardium within the CTO territory, while the remaining 27% (*n* = 15) did not show viability (Figure 1). Overall, 66% of patients had a reversible perfusion defect (ischaemia) compared to 34% without reversible perfusion defect (Figure 1). Assessment of ventricular function revealed no significant differences in left or right functional parameters (e.g., ejection fraction, end-diastolic and end-systolic volumes) between the LAD, LCx, and RCA CTO groups (Table 2). However, despite similar baseline characteristics (Table 3), patients with viable myocardium in the CTO territory exhibited significantly higher left ventricular stroke volume (LVSV: 93.6 ± 20.1 mL vs. 80.9 ± 18.4 mL, *p* = 0.039) and right ventricular stroke volume (94.1 ± 24.4 mL vs. 77.5 ± 19.9 mL, *p* = 0.025) compared to those with non-viable myocardium. However, there was no difference in the LV or RV ejection fraction between the two sub-groups (*p* = 0.052 and 0.312, respectively) (Table 4).

### 3.3. Location of CTO Lesions

In our cohort of fifty-six patients, the RCA was the most affected vessel, with thirty-two CTO lesions identified. Of these, twenty-seven occurred in individuals with right-dominant coronary circulation and were distributed as seventeen proximal, twelve mid-segment, and three distal occlusions. The LAD contained fourteen CTO lesions, evenly split between seven proximal and seven mid-segment occlusions. The LCx harboured ten CTO lesions, five of which were found in patients with left-dominant circulation. These included eight proximal and two mid-segment occlusions.

### 3.4. By-Stander Coronary Artery Disease

Several patients exhibited significant non-CTO bystander disease, characterised by moderate to severe angiographic stenosis (>70%) in the non-occluded coronary vessels. Specifically, four cases involved the RCA—two in patients with left-dominant and two with right-dominant circulation. Fourteen cases showed significant stenosis in the proximal or mid LCx artery, with ten of these occurring in individuals with left-dominant circulation. Additionally, eleven patients had significant disease in the proximal or mid segments of the LAD artery. These angiographic findings highlight the extensive coronary artery disease burden in our CTO cohort, providing insight into the location of occlusions, the prevalence of multi-vessel involvement, and the significant stenotic lesions in non-occluded vessels.

### 3.5. Myocardial Scar Burden and Perfusion Defect per CTO Territory

**Figure 2:**
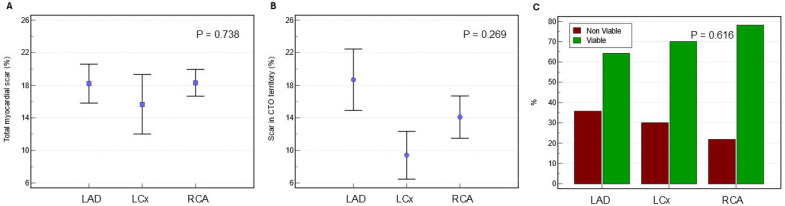
(**A**) Displays the mean (± standard deviation) proportion of total left ventricular myocardial scarring across the LAD, LCx, and RCA subgroups. (**B**) Shows the average extent (mean ± SD) of scar specifically within the CTO territory for each subgroup. (**C**) Uses bar charts to represent the percentage of patients with viable myocardium in the LAD, LCx, and RCA groups. Statistical significance was evaluated using one-way ANOVA.

Baseline symptom burden was similar among the three subgroups based on CTO territory, i.e., LAD, LCx, or RCA (Table 1). The proportion of patients exhibiting viable myocardium did not significantly differ across the three CTO territories (LAD: 64%, LCx 70%, RCA: 78%; *p* = 0.616) (Figure 2), nor did the presence of reversible myocardial ischaemia (LAD: 57%, LCx: 60%, and RCA: 69%; *p* = 0.727) (Table 2). The difference in prevalence of reversible ischaemia in non-CTO territories was not statistically significant across the three sub-groups (LAD: 36%; LCx: 60%; RCA: 53%; *p* = 0.449) (Table 2).

Quantitative assessment of myocardial scar revealed no significant differences in total left ventricular scar mass (22.6 ± 13.8 g in LAD, 22.8 ± 17.9 g in LCx, and 22 ± 13.7 g in RCA; *p* = 0.986) or total left ventricular scar percentage (18.2 ± 8.6% in LAD, 15.7 ± 11.0% in LCx, and 18.3 ± 9.1% in RCA; *p* = 0.738) across the three CTO territory sub-groups (Figure 2). Similarly, myocardial scar burden specifically within the CTO territory showed no significant variation in either absolute scar mass (8.5 ± 7.6 g, 4.4 ± 4.2 g, and 5.9 ± 7.2 g for LAD, LCx, and RCA, respectively; *p* = 0.347) or scar percentage (18.7 ± 13.6%, 9.4 ± 8.8%, and 14.1 ± 14.4% for LAD, LCx, and RCA, respectively; *p* = 0.269) (Table 2).

Analysis of perfusion metrics revealed that the perfusion defect mass within the CTO territory was significantly greater in the LAD and LCx groups compared to the RCA group (11.0 ± 3.8 g in LAD, 11.7 ± 6.8 g in LCx, and 6.7 ± 5.7 g in RCA; *p* = 0.016). However, the relative percentage of the perfusion defect within the affected territories did not differ significantly between the three sub-groups (23.7 ± 7.7% in LAD, 15.3 ± 11.4% in LCx, and 16.2 ± 14.5% in RCA; *p* = 0.162). Similarly, no significant difference was observed in the reversible perfusion defect (ischaemia) among the three sub-groups (2.5 ± 8.4 g in the LAD; 7.3 ± 6.8 g in the LCx; and 0.8 ± 8.5 g in the RCA; *p* = 0.114). When considering the total left ventricular perfusion defect mass, no significant differences were found among the LAD, LCx, and RCA sub-groups (33.1 ± 10.7 g, 30.1 ± 16.2 g, and 30.0 ± 15.4 g, respectively; *p* = 0.803). The total left ventricular reversible perfusion defect also did not vary significantly across CTO territories (10.5 ± 8.4 g in LAD, 7.2 ± 14.7 g in LCx, and 8 ± 15.8 g in RCA; *p* = 0.827) (Table 2).

### 3.6. Relationship Between Myocardial Scar Burden, Ischemia, and Viability

In the entire cohort in this study, the mean total LV scar mass was 22.3 ± 14.6 g, accounting for 17.8 ± 9.4% of the LV mass. The mean total LV perfusion defect was 30.8 ± 14.6 g, with a mean total LV reversible perfusion defect of 8.5 ± 14.1 g. Within the CTO territory, the mean proportion of scar tissue was 14.4 ± 13.7%, while the mean proportion of the reversible perfusion defect was 8.1 ± 9.6% (Table 3).

**Figure 3:**
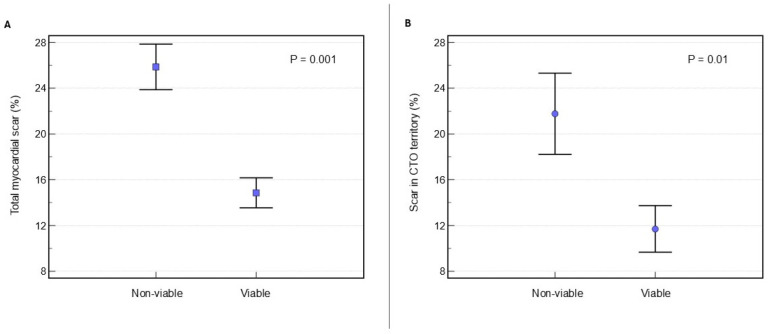
(**A**) Shows the mean (± standard deviation) proportion of the total left ventricular myocardial scar burden in the viable and non-viable subgroups. (**B**) Displays the mean (± standard deviation) proportion of the LV myocardial scarring confined to the CTO territory between the two groups. Statistical significance was assessed using an independent *t*-test.

**Figure 4:**
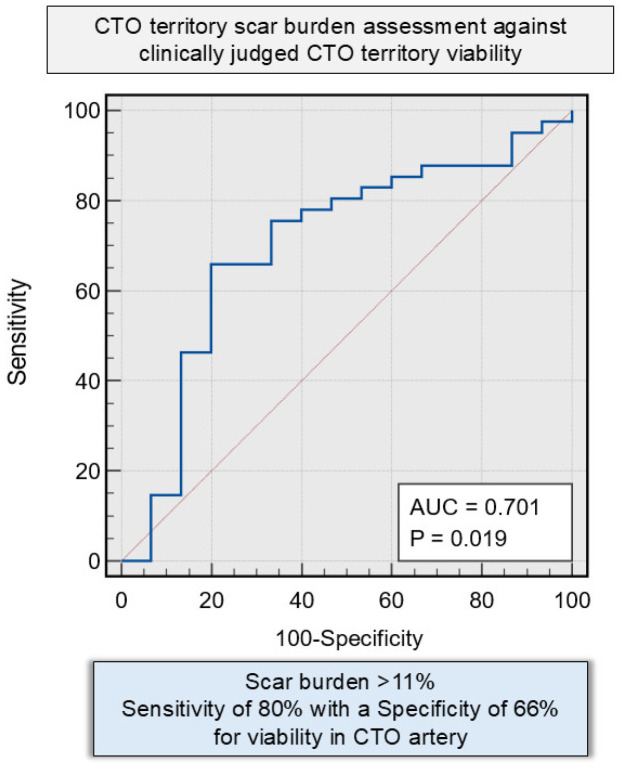
Receiver operating characteristic (ROC) analysis evaluating the discriminative performance of CTO territory scar burden in identifying patients with clinically judged non-viable CTO-territory myocardium. The blue curve represents the ROC curve showing the relationship between sensitivity and 1-specificity across different scar burden thresholds, while the red diagonal line represents the line of no discrimination (area under the curve [AUC = 0.5]). The AUC was 0.701 (95% CI 0.54–0.87; *p* = 0.019), indicating modest discriminatory ability.

Patients exhibiting non-viable myocardium in the CTO territory demonstrated a significantly higher total LV scar burden, both in terms of absolute scar mass (32.3 ± 12.8 g vs. 18.7 ± 13.5 g; *p* = 0.002) and as a proportion of total LV mass (25.9 ± 7.5% vs. 14.9 ± 8.3%; *p* = 0.001), compared to patients with viable myocardium (Figure 3 and Table 4). Furthermore, the localised LV scar mass within the CTO territory was substantially greater in the non-viable group (9.4 ± 6.5 g) than in the viable group (5.1 ± 6.9 g; *p* = 0.046). Similarly, the proportion of LV scar within the CTO territory was significantly higher (*p* = 0.01) in the non-viable group (21.8 ± 13.3%) compared to the viable group (11.7 ± 12.8%), highlighting a direct association between myocardial non-viability and regional fibrosis (Figure 3 and Table 4). The prevalence of overall left ventricular perfusion defect was similar between the two sub-groups (32.1 ± 10.3 g in non-viable vs. 30.3 ± 15.8 g in viable; *p* = 0.687). However, a notable distinction emerged in the pattern of ischaemia. Patients with viable myocardium had a significantly higher total LV burden of reversible perfusion defects (ischaemia) in non-scarred regions (11.6 ± 14.3 g in the viable group vs. 0.2 ± 9.3 g in the non-viable group; *p* = 0.005). They also showed a greater percentage of reversible perfusion defects (10.3 ± 10.3% in viable vs. 2.3 ± 2.7% in non-viable; *p* = 0.005), reflecting a higher level of inducible ischaemia (Table 4). Furthermore, a total myocardial scar burden threshold of 11.18% demonstrated diagnostic utility in predicting myocardial non-viability, yielding a sensitivity of 80% and a specificity of 65.85% (AUC = 0.701 [95% CI 0.54–0.87]; *p* = 0.019) (Figure 4).

## 4. Discussion

This study clarifies the myocardial pathophysiology of CTOs and establishes the additive power of multiparametric CMR beyond coronary angiography [14,15,33]. CTO territories proved heterogeneous: 27% of patients harboured negligible or limited viable myocardium—a subgroup who benefits from CTO revascularisation is uncertain—whereas the remaining 73% preserved substantial viability, with two-thirds of those segments (66%) demonstrating reversible ischaemia suggesting a potentially recoverable phenotype. These data highlight multiparametric CMR as a comprehensive phenotyping tool that may inform shared decision-making regarding CTO revascularisation.

A scar burden threshold of approximately 11% in a CTO territory emerged as a potential marker distinguishing viable from non-viable myocardium, consistent with the concept that extensive fibrosis limits the likelihood of functional recovery. This metric could help guide patient selection for CTO interventions by combining anatomical and functional assessments. Patients with viable myocardium exhibited lower scar burden and increased reversible ischaemia. While their ejection fractions were higher compared to the non-viable group (49.1 ± 10.3% vs. 43.1 ± 8.8%, *p* = 0.052), this difference was not statistically significant. Additionally, there was no significant difference in LV end-diastolic volumes between the two groups. However, the viable group had significantly higher stroke volumes, suggesting a preserved subclinical contractile reserve. These findings align with prior studies showing that myocardial viability predicts favourable remodelling after CTO-PCI [33,34,35,36,37,38,39]. Importantly, viability was observed across all vascular territories, reinforcing the need for individualised, physiology-driven assessment rather than reliance on anatomical location alone. This threshold is intended to complement segmental transmural LGE assessment by summarising overall CTO-territory scar burden at the patient level, rather than serve as a stand-alone clinical cut-off.

Stress perfusion assessment further delineated the ischaemic landscape within CTO territories. While the absolute perfusion defect mass was greater in the LAD and LCx distributions, relative perfusion burden did not differ significantly by vascular territory. These perfusion findings further support the utility of CMR in identifying patients with significant, yet potentially reversible, ischaemic burden.

The observed differences in right ventricular volumes and stroke volume between viability groups were not a prespecified endpoint and may reflect differences in body size and/or loading conditions.

The limited survival benefit observed in RCTs of CTO-PCI—among other limitations—may reflect a fundamental misalignment between the trial populations and real-world patients [6,8,38,39,40,41,42]. Robust RCT data evaluating functional imaging prior to CTO PCI remains scarce or lacking, relying primarily on anatomical criteria that do not adequately capture myocardial viability or ischaemic burden [7,29]. Stress perfusion CMR with LGE, through myocardial characterisation, offers a more precise tool to better phenotype and guide treatment strategy in this very high risk population [15,28].

**Figure 5:**
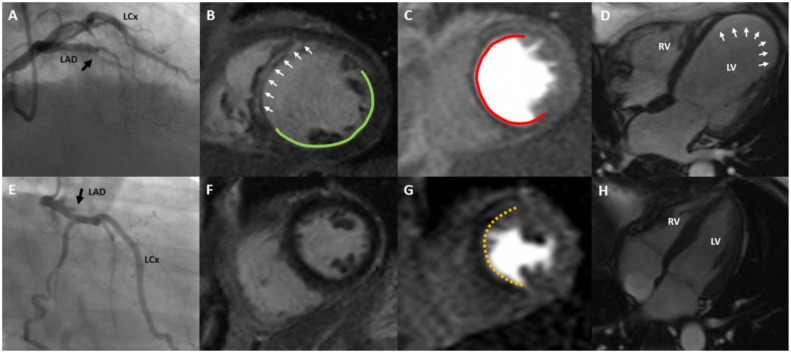
Two case examples of LAD CTO with CMR-based myocardial characterisation. (**A**) A coronary angiography image in the right anterior oblique (RAO) cranial view, showing a chronic total occlusion (CTO) in the left anterior descending (LAD) artery, marked by a black arrow. (**B**) A mid-left ventricular (LV) short-axis T1 mapping image highlighting a large myocardial scar occupying more than 50% of the wall thickness. The scarred region (LV septum) is indicated by white arrows, while the normal myocardium is outlined in green. (**C**) A single frame from a dynamic stress perfusion scan (short-axis, mid-ventricle) revealing a fixed perfusion defect across the LAD territory in the LV septum, outlined in red. (**D**) An apical four-chamber (A4C) cine view showing thinning of the LV apex indicated by white arrows. (**E**) A postero-anterior caudal angiographic view displaying a proximal LAD CTO, marked with a black arrow. (**F**) A mid-ventricular short-axis T1 map showing no evidence of scar in the LV myocardium. (**G**) A frame from a mid-ventricular short-axis stress perfusion study showing a stress-induced perfusion defect in the septal and anterior segments of the LV, marked by a yellow outline. (**H**) An A4C cine image of the LV, providing structural visualisation without additional findings noted.

Our findings reinforce the existence of two biologically distinct phenotypes within the CTO population (Figure 5). “Responders” manifest preserved myocardial viability with reversible ischaemia; in a quantitative PET cohort, successful CTO-PCI has been associated with large reductions in perfusion-defect burden and a 66% lower risk of death or non-fatal infarction at long-term follow-up, as shown by quantitative PET imaging [43]. “Non-responders”, by contrast, exhibit fixed defects devoid of viability, and CMR series consistently demonstrate absent contractile recovery and uncertain incremental clinical benefit after technically successful recanalisation [36,37].

These converging data position stress-CMR as a mechanistic gatekeeper that may help inform revascularisation towards viable, ischaemic myocardium while identifying non-viable territories where benefit from CTO-PCI is uncertain and where optimised medical therapy and/or trials may be appropriate, thereby sharpening patient selection and minimising futile intervention.

### Limitations

This study has several limitations that should be considered. The sample size of 56 patients may have limited the statistical power to detect smaller but potentially clinically significant differences between the viable and non-viable myocardium groups or across the different CTO territories. The modest sample size and single-centre design, despite being detailed, may constrain the generalisability of the findings to broader populations. Referral bias is another limitation, as the cohort consisted of patients referred for CMR, likely representing a subgroup with an intermediate to high pre-test probability of myocardial viability or planned PCI. This could overrepresent individuals with suspected myocardial abnormalities or those being considered for revascularisation, thereby not reflecting the broader CTO patient population, including asymptomatic or conservatively managed individuals. Exclusion criteria related to CMR contraindications may have further skewed cohort characteristics. The absence of long-term clinical follow-up restricts the study’s ability to assess PCI outcomes, such as improvements in LV function, symptom burden, or survival, limiting the prognostic relevance of the imaging-based phenotypic classifications. Additionally, the criteria used to define myocardial viability and non-viability based on LGE thresholds (e.g., <50% vs. >50% transmurality), while consistent with established conventions, may not reliably predict functional recovery in the context of CTO, where prolonged myocardial stunning or hibernation may occur. The presence of myocardial stunning or hibernation complicates the interpretation of LGE findings, highlighting the need for studies incorporating follow-up functional data to validate these surrogate markers. Finally, variability in individual hemodynamic responses to adenosine poses a limitation for stress perfusion assessment, as suboptimal hyperaemic responses could compromise the accuracy of perfusion measurements. Perfusion defects were assessed visually and translated into a mass by segmental summation; quantitative perfusion mapping was not performed, and interobserver reproducibility was not formally assessed. Multivessel coronary disease and overlap between coronary perfusion territories may confound attribution of inducible ischaemia to the CTO vessel; given the limited sample size, we did not perform sensitivity analyses excluding severe bystander stenoses. Antianginal medication at the time of CMR was not consistently captured and may have influenced ischaemic burden.

Future investigation should first expand the observational evidence base by embedding stress-CMR into large, prospective registries—so that quantitative viability and ischaemia metrics can be linked to long-term, hard endpoints (death, myocardial infarction, and heart failure admission) captured through national health records. As stress-CMR becomes more widely available and affordable, and given its ability to provide precise and clinically meaningful information, it should be considered a first-line diagnostic tool over echocardiography in CTO. Such real-world data will refine imaging thresholds, identify responder phenotypes and generate hypothesis-driven risk models. The next step is a pragmatic, multicentre randomised trial in which CMR-defined responders are allocated to PCI or optimal medical therapy, while non-responders are randomised to contemporary pharmacotherapy alone; powered for all-cause mortality and non-fatal MI. Finally, parallel cost-effectiveness analyses are crucial to fully evaluate the economic benefits of adopting a “CMR-first” approach in CTO management. Building on existing evidence from stable coronary disease, these analyses should consider both upfront costs and long-term savings from reduced hospitalisations, invasive procedures, and adverse events across diverse healthcare systems. Such comprehensive data will guide policymakers and clinicians, support reimbursement frameworks, and encourage broader guideline implementation.

## 5. Conclusions

In patients with CTO, up to 27% harbour no—or only limited—viability, whereas the remaining patients have preserved viable myocardium that typically exhibits clinically relevant reversible ischaemia and therefore stands to benefit from revascularisation. These findings support the use of multiparametric CMR to phenotype CTO territories prior to considering CTO-PCI.

## Figures and Tables

**Table 1:** Baseline demographics and clinical characteristics stratified by CTO location.

CTO Lesions	All (*n* = 56)	LAD (*n* = 14)	LCx (*n* = 10)	RCA (*n* = 32)	* *p*-Value
Gender (male)	49 (88%)	12 (86%)	8 (80%)	29 (91%)	0.656
Age (years)	70.7 ± 10.2	70.4 ± 10	70.1 ± 9.5	71.1 ± 10.4	0.955
Height (cm)	171.8 ± 8.9	170.8 ± 10.3	171.3 ± 7.4	172.3 ± 8.5	0.865
Weight (kg)	86.8 ± 15.7	82.7 ± 18.7	84.9 ± 11	89.2 ± 15.1	0.419
Haemoglobin (g/L)	134 ± 21.5	131.6 ± 19.3	138.9 ± 26.8	133.5 ± 20.4	0.71
eGFR (mL/min/1.73 m^2^)	70.5 ± 19.1	69.9 ± 21	70.5 ± 25.7	70.8 ± 15.5	0.98
CKD	24 (43%)	4 (29%)	4 (40%)	16 (50%)	0.41
Dyslipidaemia	29 (52%)	9 (64%)	4 (40%)	16 (50%)	0.49
Diabetes Mellitus	20 (36%)	4 (29%)	2 (20%)	14 (44%)	0.331
Hypertension	27 (48%)	7 (50%)	3 (30%)	17 (53%)	0.451
Smoking history	37 (66%)	10 (71%)	7 (70%)	20 (63%)	0.815
Atrial fibrillation	8 (14%)	3 (21%)	1 (10%)	4 (13%)	0.678
CVA	4 (7%)	1 (7%)	0 (0%)	3 (9%)	0.617
Previous MI	43 (77%)	11 (79%)	5 (50%)	27 (84%)	0.08
VT	7 (13%)	0 (0%)	1 (10%)	6 (19%)	0.21
CCS Class **	3 ± 0.7	2.8 ± 0.8	2.9 ± 0.7	3.1 ± 0.7	0.35
NYHA Class ***	2.2 ± 1	2.4 ± 0.8	1.7 ± 1	2.3 ± 1	0.848

All data are presented as mean ± SD or *n* (%). CTO, chronic total occlusion; LAD, left anterior descending; LCx, left circumflex; RCA, right coronary artery; eGFR, estimated glomerular filtration rate; CKD, chronic kidney disease; CVA, cerebrovascular accident; MI, myocardial infarction; VT, ventricular tachycardia. * Difference between the CTO territory groups using one-way analysis of variance (ANOVA). ** Canadian Cardiovascular Society. *** New York Heart Association.

**Table 2:** Cardiovascular magnetic resonance-derived imaging characteristics across different CTO territories.

CTO Lesions	All (*n* = 56)	LAD (*n* = 14)	LCx (*n* = 10)	RCA (*n* = 32)	* *p*-Value
Volumetric CMR assessment					
Left ventricular end-diastolic volume (mL)	196.1 ± 51.3	196.4 ± 33.7	217 ± 69.8	189.4 ± 49	0.343
Left ventricular end-systolic volume (mL)	105.8 ± 43.1	111.5 ± 31.5	122.3 ± 64.3	98.2 ± 37	0.27
Left ventricular stroke volume (mL)	90.2 ± 20.4	84.9 ± 12.9	94.7 ± 21.7	91.1 ± 22.2	0.488
Left ventricular ejection fraction (%)	47.5 ± 10.2	44.1 ± 8.1	46.9 ± 14	49.1 ± 9.3	0.317
Left ventricular end-diastolic mass (g)	137.4 ± 34	127.9 ± 21.9	149.5 ± 44.6	137.7 ± 33.3	0.322
Right ventricular end-diastolic volume (mL)	154.5 ± 34.7	146.3 ± 25.7	158 ± 39.5	156.9 ± 35.9	0.609
Right ventricular end-systolic volume (mL)	64.8 ± 20.3	59.5 ± 20	68.3 ± 21.9	66.1 ± 19.5	0.518
Right ventricular stroke volume (mL)	89.6 ± 24.4	86.8 ± 18.3	89.8 ± 23	90.8 ± 27	0.883
Right ventricular ejection fraction (%)	58.2 ± 9.5	59.9 ± 10.2	56.9 ± 5.6	57.9 ± 9.9	0.717
Myocardial scar and perfusion metrics					
Total LV scar (g)	22.3 ± 14.6	22.6 ± 13.8	22.8 ± 17.9	22 ± 13.7	0.986
Total LV scar (%)	17.8 ± 9.4	18.2 ± 8.6	15.7 ± 11	18.3 ± 9.1	0.738
Total LV perfusion defect (g)	30.8 ± 14.6	33.1 ± 10.7	30.1 ± 16.2	30 ± 15.4	0.803
Total LV reversible perfusion defect (g)	8.5 ± 14.1	10.5 ± 8.4	7.2 ± 14.7	8 ± 15.8	0.827
LV scar mass in CTO territory (g)	6.3 ± 7	8.5 ± 7.6	4.4 ± 4.2	5.9 ± 7.2	0.347
LV scar in CTO territory (%)	14.4 ± 13.7	18.7 ± 13.6	9.4 ± 8.8	14.1 ± 14.4	0.269
CTO territory perfusion defect (g)	8.7 ± 6	11 ± 3.8	11.7 ± 6.8	6.7 ± 5.7	0.016
CTO territory perfusion defect (%)	17.9 ± 13	23.7 ± 7.7	15.3 ± 11.4	16.2 ± 14.5	0.162
Reversible perfusion defect in CTO territory (g)	2.4 ± 8.6	2.5 ± 8.4	7.3 ± 6.8	0.8 ± 8.5	0.114
Reversible perfusion defect in CTO territory (%)	36 (64%)	8 (57%)	6 (60%)	22 (69%)	0.727
Reversible perfusion defect in non-CTO territory (%)	28 (50%)	5 (36%)	6 (60%)	17 (53%)	0.449
CTO territory viability (%)	41 (73%)	9 (64%)	7 (70%)	25 (78%)	0.616

All data are presented as mean ± SD or *n* (%). CMR, cardiovascular magnetic resonance; LV, left ventricle; CTO, chronic total occlusion; LAD, left anterior descending; LCx, left circumflex; RCA, right coronary artery. * Difference between the CTO territory groups using one-way analysis of variance (ANOVA).

**Table 3:** Baseline patient characteristics comparing viable versus non-viable myocardial subgroups.

	All (*n* = 56)	Non-Viable (*n* = 15)	Viable (*n* = 41)	* *p*-Value
Gender (male)	49 (88%)	11 (73%)	38 (93%)	0.054
Age (years)	70.7 ± 10.2	74.3 ± 6.8	69.4 ± 10.9	0.113
Height (cm)	171.8 ± 8.9	167.8 ± 9.8	173.2 ± 8	0.045
Weight (kg)	86.8 ± 15.7	86.7 ± 18.7	86.8 ± 14.5	0.987
Body mass index (kg/m^2^)	29.2 ± 5.4	30.1 ± 5.2	28.9 ± 5.4	0.480
Haemoglobin (g/L)	134 ± 21.5	131 ± 25.8	135.1 ± 19.6	0.540
eGFR (mL/min/1.73 m^2^)	70.5 ± 19.1	64.1 ± 20.3	72.9 ± 18.1	0.133
CKD	24 (43%)	7 (47%)	17 (41%)	0.733
Dyslipidaemia	29 (52%)	5 (33%)	24 (59%)	0.098
Diabetes mellitus	20 (36%)	6 (40%)	14 (34%)	0.692
Hypertension	27 (48%)	11 (73%)	16 (39%)	0.023
Smoking history	37 (66%)	9 (60%)	28 (68%)	0.570
Atrial fibrillation	8 (14%)	4 (27%)	4 (10%)	0.113
CVA	4 (7%)	0 (0%)	4 (10%)	0.217
Previous MI	43 (77%)	11 (73%)	32 (78%)	0.717
VT	7 (13%)	0 (0%)	7 (17%)	0.090
CCS class **	3 ± 0.7	3.1 ± 0.8	2.9 ± 0.7	0.520
NYHA class ***	2.2 ± 1	2.5 ± 1	2.1 ± 1	0.288

All data are presented as mean ± SD or *n* (%). eGFR, estimated glomerular filtration rate; CKD, chronic kidney disease; CVA, cerebrovascular accident; MI, myocardial infarction; VT, ventricular tachycardia. * Difference between viable and non-viable groups using independent samples *t*-test. ** Canadian Cardiovascular Society. *** New York Heart Association.

**Table 4:** Cardiovascular magnetic resonance imaging characteristics in relation to myocardial viability status.

	All (*n* = 56)	Non-Viable (*n* = 15)	Viable (*n* = 41)	* *p*-Value
Volumetric CMR assessment				
Left ventricular end-diastolic volume (mL)	196.1 ± 51.3	192.9 ± 48.8	197.2 ± 52.1	0.783
Left ventricular end-systolic volume (mL)	105.8 ± 43.1	112 ± 39.7	103.6 ± 44.1	0.528
Left ventricular stroke volume (mL)	90.2 ± 20.4	80.9 ± 18.4	93.6 ± 20.1	0.039
Left ventricular ejection fraction (%)	47.5 ± 10.2	43.1 ± 8.8	49.1 ± 10.3	0.052
Left ventricular end-diastolic mass (g)	137.4 ± 34	126.3 ± 29.2	141.4 ± 34.8	0.145
Right ventricular end-diastolic volume (mL)	154.5 ± 34.7	130.7 ± 33	163.2 ± 31	0.001
Right ventricular end-systolic volume (mL)	64.8 ± 20.3	53.1 ± 19.4	69.1 ± 18.9	0.009
Right ventricular stroke volume (mL)	89.6 ± 24.4	77.5 ± 19.9	94.1 ± 24.4	0.025
Right ventricular ejection fraction (%)	58.2 ± 9.5	60.4 ± 9.9	57.4 ± 9.2	0.312
Myocardial scar and perfusion metrics				
Total LV scar (g)	22.3 ± 14.6	32.3 ± 12.8	18.7 ± 13.5	0.002
Total LV scar (%)	17.8 ± 9.4	25.9 ± 7.5	14.9 ± 8.3	0.001
Total LV perfusion defect (g)	30.8 ± 14.6	32.1 ± 10.3	30.3 ± 15.8	0.687
Total LV reversible perfusion defect (g)	8.5 ± 14.1	0.2 ± 9.3	11.6 ± 14.3	0.005
LV scar mass in CTO territory (g)	6.3 ± 7	9.4 ± 6.5	5.1 ± 6.9	0.046
LV scar in CTO territory (%)	14.4 ± 13.7	21.8 ± 13.3	11.7 ± 12.8	0.01
CTO territory perfusion defect (g)	8.7 ± 6	9 ± 5.1	8.6 ± 6.2	0.854
CTO territory perfusion defect (%)	17.9 ± 13	17 ± 8.3	18.2 ± 14.3	0.75
CTO territory reversible perfusion defect (g)	2.4 ± 8.6	0.5 ± 6.7	3.5 ± 8.9	0.134
CTO territory reversible perfusion defect (%)	8.1 ± 9.6	2.3 ± 2.7	10.3 ± 10.3	0.005

All data are presented as mean ± SD or *n* (%). CMR, cardiovascular magnetic resonance; LV, left ventricle; CTO, chronic total occlusion. * Difference between viable and non-viable groups using independent samples *t*-test.

## Data Availability

The datasets generated and analysed during the current study are not publicly available. Access to the raw images of patients is not permitted since specialised post-processing imaging-based solutions can identify the study patients in the future. Data are available from the corresponding author upon reasonable request.

## Abbreviations

AHA	American Heart Association
CTO	chronic total occlusion
CMR	cardiac magnetic resonance
LV	left ventricle
LGE	late gadolinium enhancement
LAD	left anterior descending
LCx	left circumflex
NYHA	New York Heart Association
PCI	percutaneous coronary intervention
RCT	randomised controlled trials
RCA	right coronary artery
AUC	area under the curve
bSSFP	balanced steady-state free precession
CKD	chronic kidney disease
CCS	Canadian Cardiovascular Society
ECG	electrocardiogram
EDV	end-diastolic volume
EF	ejection fraction
eGFR	estimated glomerular filtration rate
ESV	end-systolic volume
GDMT	guideline-directed medical therapy
GRAPPA	generalised autocalibrating partially parallel acquisitions
MI	myocardial infarction
ROC	receiver operating characteristic
RV	right ventricle
RVEF	right ventricular ejection fraction
SV	stroke volume
